# Development of Trans-1,4-Polyisoprene Shape-Memory Polymer Composites Reinforced with Carbon Nanotubes Modified by Polydopamine

**DOI:** 10.3390/polym14010110

**Published:** 2021-12-29

**Authors:** Chuang Zhang, Long Li, Yuanhang Xin, Jiaqi You, Jing Zhang, Wanlu Fu, Na Wang

**Affiliations:** 1 Liaoning Provincial Key Laboratory for Preparation and Application of Special Functional Materials, Shenyang University of Chemical Technology, Shenyang 110142, China; iamzhangchuang@163.com (C.Z.); lilong@syuct.edu.cn (L.L.); iamxinyuanhang@163.com (Y.X.); iamyoujiaqi@163.com (J.Y.); zhangjingcszx@syuct.edu.cn (J.Z.); 2 Shenyang Research Institute of Industrial Technology for Advanced Coating Materials, Shenyang 110142, China; 13464038746@163.com

**Keywords:** trans-1,4-polyisoprene, carbon nanotubes, mechanical properties, thermal stability, shape-memory properties

## Abstract

In this study, which was inspired by mussel-biomimetic bonding research, carbon nanotubes (CNTs) were interfacially modified with polydopamine (PDA) to prepare a novel nano-filler (CNTs@PDA). The structure and properties of the CNTs@PDA were studied using scanning electron microscopy (SEM), Fourier transform infrared spectroscopy (FTIR), Raman spectroscopy, X-ray photoelectron spectroscopy (XPS), and thermogravimetric analysis (TGA). The CNTs and the CNTs@PDA were used as nanofillers and melt-blended into trans-1,4 polyisoprene (TPI) to create shape-memory polymer composites. The thermal stability, mechanical properties, and shape-memory properties of the TPI/CNTs and TPI/CNTs@PDA composites were systematically studied. The results demonstrate that these modifications enhanced the interfacial interaction, thermal stability, and mechanical properties of TPI/CNTs@PDA composites while maintaining shape-memory performance.

## 1. Introduction

Shape-memory polymer (SMP) refers to a type of smart polymer that can return to its initial shape from a temporary deformed shape under external stimuli such as heat, light, electricity, magnetism, pH value, metal ions, and many other physical and chemical stimuli [1,2,3]. Because of their unique shape-recovery function, SMPs have broad application prospects in many fields, including biological medical devices, aerospace engineering, textiles, and anti-counterfeiting [4,5,6]. Thermally induced SMP is an important branch of shape-memory polymer stimulation that has been widely studied and has many industrial applications [7,8,9]. As an important component of thermally induced SMPs, trans-1,4-polyisoprene (TPI) and its composites have been used in biomedical and protective equipment applications due to their fast deformation speed, high recovery rate, and high recovery accuracy [10]. However, poor thermal stability and weak mechanical properties limit the potential of TPI shape-memory polymers.

In recent years, researchers have found that the shape-memory and mechanical properties of shape-memory polymers can be effectively enhanced by integrating nano-fillers into a polymer’s matrix [11,12,13]. Carbon nanotubes (CNTs) are a new type of one-dimensional nanomaterial where the carbon atoms in the tube walls form a hexagonal network structure due to *sp^2^* hybridization; the basic network is the same as that of graphene. The C=C covalent bond formed by *sp^2^* hybridization is one of the strongest covalent bonds in nature, so carbon nanotubes have extremely high mechanical properties [14,15]. The elastic modulus of CNTs can reach 1TPa, which is almost the same as that of diamond and about five times that of steel [16]. However, due to their high cohesion, CNTs are prone to form intertangled cluster structures, which hinders their effective dispersion in a TPI matrix [17]. Therefore, identifying a method that will promote the uniform dispersion of CNTs in SMPs is of great importance to expand its potential applications [18]. CNTs can be functionalized by covalent and noncovalent modifications [19,20]. Of the two, covalent functionalization is the most common form of modification of CNTs, and it is also one of the most destructive methods when it comes to the CNTs’ structure. Conversely, the noncovalent modification conditions of organic polymer coatings is mild, making it an ideal modification method [21,22,23]. In addition, previous research has shown that inorganic nanomaterials modified with organic polymers may improve the compatibility and interface interaction between the nanoparticles and the matrices, allowing them to achieve better performance, as compared to that of other common composite materials [24,25,26,27].

Dopamine molecules derived from biomimetic mussels have shown strong adhesion ability [28,29]. Messersmith et al. found that dopamine could be oxidized under weak alkaline conditions, forming polydopamine (PDA) [30]. PDA can be added to almost any material and can form a thick coating on its surface because of its strong adhesion [31,32,33]. More importantly, since the surface of the PDA contains a large number of chemically active groups [34,35], the coating could effectively reduce the agglomeration of the nanofiller itself, which could further improve the dispersion of nanofillers in shape-memory polymer composites. In addition, the reaction conditions of PDA modification in nanofillers are mild (i.e., no strong acid or strong oxidant has to be used), and the surface and length of nanofillers are not damaged, indicating its potential for further development [36,37]

In this study, CNTs were modified with noncovalent bonds using oxidative autopolymerization of dopamine, and CNTs@PDA nano-fillers were prepared to improve the dispersion of CNTs. In addition, TPI shape-memory polymer composites were prepared using a melt-blending method with TPI as the matrix and CNTs@PDA as the filler. The mechanical properties, thermal stability, and shape-memory properties of TPI shape-memory polymer composites were studied and compared with neat TPI and a TPI/CNTs composite.

## 2. Materials and Methods

### 2.1. Materials

The TPI was provided by Qingdao Dipai New Material Co., Ltd., Beijing, China. Carbon nanotubes (CNTs, multi-walled, diameter of 20–30 nm and length of 1–3 μm) were purchased from Suzhou Hengqiu Technology Co., Ltd., Suzhou, China. Dopamine hydrochloride was provided by Shanghai Macklin Biochemical Co., Ltd., Shanghai, China. Tris (hydroxymethyl) aminomethane (Tris) was purchased from Tianjin Damao Chemical Reagent Factory, Tianjin, China. Sulfur, zinc oxide (ZnO), stearic acid (SA), N-Isopropyl-N’-phenyl-4-phenylenediamin (antioxidant 4010NA), and N-(Oxidiethylene)-2-benzothiazolyl sulfenamide (accelerator NOBS) were provided by Shanghai Chengjin Chemicals Co., Ltd., Shanghai, China, and utilized without further refinement.

### 2.2. Preparation of TPI/CNTs@PDA Composites

**Figure 1:**
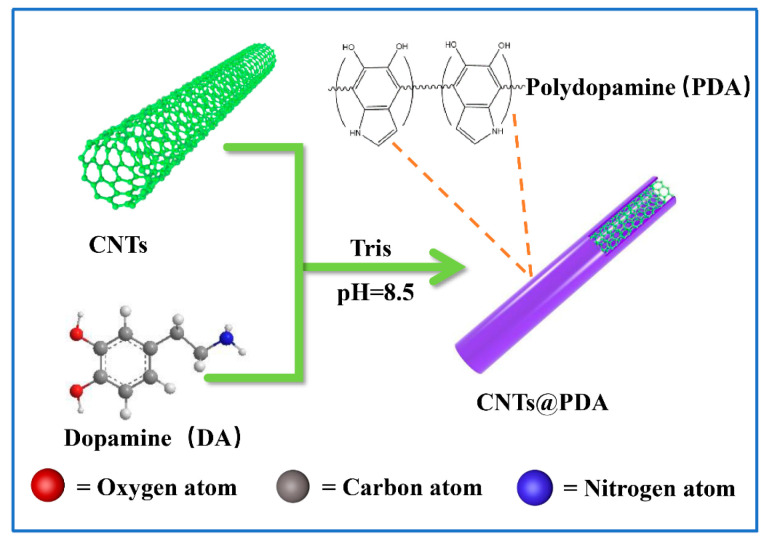
The schematic of preparation process of CNTs@PDA.

First, 600 mg of CNTs were added to 900 mL of deionized water. After ultrasonic dispersion for 30 min, 360 mg of dopamine was added under magnetic stirring, and the solution was stirred at room temperature for 20 min. Then, 100 mL Tris (1.21 g) aqueous solution was added, the temperature was adjusted to 60 °C, and the stirring continued for 8 h. The final product was centrifuged several times (3500× *g* r/min) and washed with deionized water until the filtrate was colorless. Then, it was dried in a vacuum oven at 60 °C for 24 h to obtain the CNTs@PDA nano-filler. The preparation process of the CNTs@PDA nano-filler is shown in Figure 1.

The prepared CNTs@PDA nano-filler was melt-blended with TPI in an open mill at 70 °C for 15 min. TPI composites were prepared by adding 2.0 phr (parts per hundred rubber) SA, 4.0 phr ZnO, 2.0 phr 4010NA, 1.2 phr NOBS, and 1.5 phr sulfur along with CNTs@PDA (0 phr, 0.6 phr, 1.2 phr, 1.8 phr, 2.4 phr, and 3.0 phr, respectively) to the TPI matrix. After standing at room temperature for 24 h, the composite was hot pressed in a vacuum at 5 MPa and 150 °C for 10 min to form 2 mm thick sheets. In addition, the control group was created according to the same method yielding 0.6 phr TPI/CNTs composite material that contained exclusively CNTs.

### 2.3. Characterizations

Fourier transform infrared (FTIR) spectra were recorded using a Fourier spectrometer (Nicolet MAGNA-IR560) to characterize the chemical structures of CNTs and CNTs@PDA.

Scanning electron microscopy (SEM) was used to analyze the microstructures of the nanocomposite samples. The SEM micrographs were obtained by JSM-6360LV (Japan Jeol).

Raman spectra were obtained using a multichannel confocal spectrometer (HORIBA Scientific LabRAM HR Evolution) with a laser wavelength of 535 nm.

X-ray diffraction (XRD) analysis was recorded by D & Advance (Germany Bruker) with CuK_α_ radiation (λ = 0.154 nm), and the diffraction angle was decreased from 5° to 80° with a scanning rate of 3°/min.

X-ray photoelectron spectroscopy (XPS) was performed by an X-ray photoelectron spectrometer (KRATOS, Axis UltraDLD). 

Thermogravimetric analysis (TGA) was performed on a STA449C (Germany Netzsch). Under a nitrogen environment, the range of test temperatures was from 40 to 800 °C at a heating rate of 10 °C/min.

Dynamic mechanical analysis (DMA) was carried out by using a DMAQ800 (American TA). The frequency and heating rate were set at 1 Hz and 3 °C/min from −75 to 50 °C.

Differential scanning calorimetry (DSC) analysis was used by a DSCQ200 (American TA) under a nitrogen atmosphere. Samples with a mass of 8–10 mg were maintained at 100 °C for 5 min then cooled to −80 °C at 10 °C/min for 5 min, and samples were heated to 100 °C at 10 °C/min. The degree of crystallinity (*X_c_*) for each portion of the sample was calculated by the following equation:

(1) $$X_{c}=\frac{\Delta H_{m}}{\Delta H_{m*}}\times 100\%,$$

where $\Delta H_{m}$ and $\Delta H_{m*}$ are the melting enthalpy of a certain polymer portion and that of pure polymer (ca. 186.8 J/g for TPI), respectively [38].

The shape-memory properties of the composites were analyzed by DMA. Firstly, the samples were equilibrated at 60 °C and then stretched at a stress of 0.4 MPa. Secondly, the sample was maintained at a constant stress and cooled down to −10 °C quickly, equilibrated at this temperature for 5 min, and then the stress was removed. In the last step, the samples were heated to 60 °C and equilibrated. The fixing ratio (*R_f_*) and recovery ratio (*R_r_*) were used to calculate the following formulas.

(2) $$R_{f}\left(X\to Y\right)=\frac{\varepsilon_{Y}-\varepsilon_{X}}{\varepsilon_{\left(Y,load\right)}-\varepsilon_{X}}\times 100\%,$$

(3) $$R_{r}\left(X\to Y\right)=\frac{\varepsilon_{Y}-\varepsilon_{\left(X,rec\right)}}{\varepsilon_{Y}-\varepsilon_{X}}\times 100\%.$$

## 3. Results and Discussion

### 3.1. Characterization of CNTs and CNTs@PDA 

**Figure 2:**
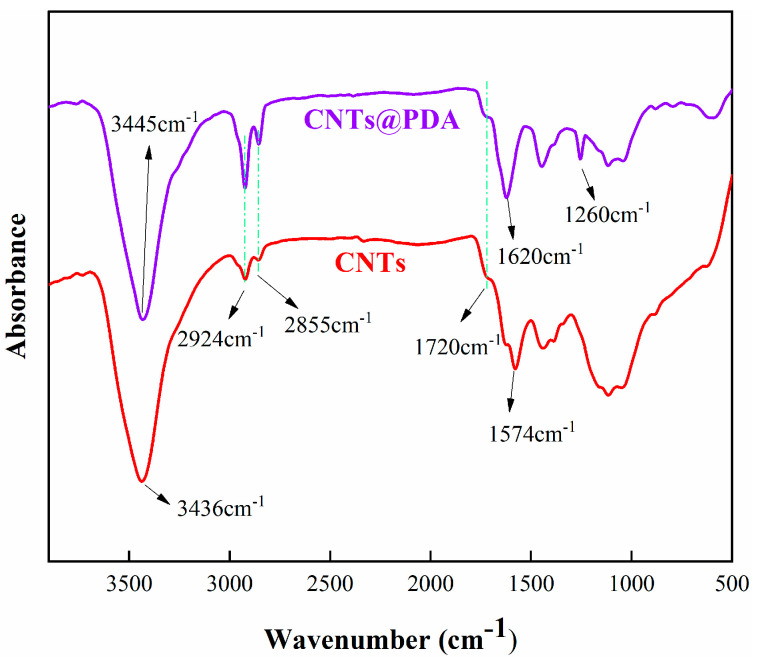
FTIR spectra of CNTs and CNTs@PDA.

Figure 2 shows the FTIR spectra of CNTs and CNTs@PDA samples. For the spectra of the original CNTs, obvious bands were observed at 3436 and 1574 cm^−1^, which may have been caused by the adsorption of -OH groups on CNTs and the C=C stretching vibration of CNTs while at 1720 cm^−1^, and the corresponding stretching vibration peak of C=O was observed. The absorption peaks at 2924 and 2855 cm^−1^ corresponded to the stretching vibration of aliphatic C-H, and the intensity of the absorption peaks of CNTs@PDA increases significantly, which is caused by the increase in the surface carbon content of CNTs after coating. In addition, after the auto-oxidation polymerization of dopamine, PDA was formed, which had a strong absorption peak at 1260 and 1620 cm^−1^, which was caused by the stretching vibration of C-O and the aromatic ring, respectively. There was a wide peak at 3445 cm^−1^, which was caused by the combination of the two characteristic peaks resulting from the abundant N-H and -OH on the PDA. These results clearly indicate that the CNTs were successfully functionalized by the PDA.

**Figure 3:**
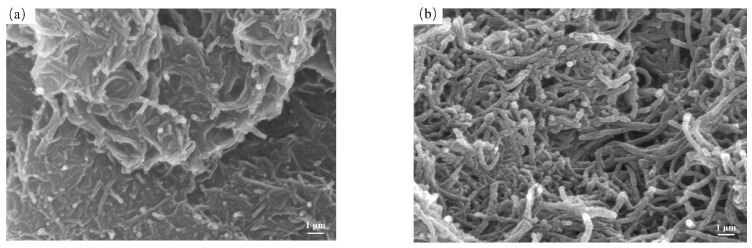
SEM images of (**a**) CNTs and (**b**) CNTs@PDA.

In order to explore the morphological changes of CNTs before and after modification, the surface morphology of CNTs was characterized by SEM. The SEM images of CNTs and CNTs@PDA are shown in Figure 3. As can be seen from the figure, the surface of unmodified CNTs was relatively smooth and clear in outline, while the surface of the CNTs@PDA had an obvious coating layer that was dense and complete in structure, and the diameter of the tube was increased relative to that of the CNTs. The unmodified CNTs also had a lot of entanglement and agglomeration. Comparatively, the dispersibility of the CNTs@PDA was significantly improved due to the PDA coating and its large number of active surface groups. In addition, the PDA coating formed a physical steric hindrance between the CNTs, which effectively prevented the agglomeration of the CNTs. Therefore, we determined that the PDA had both successfully coated the CNTs and provided additional benefits.

**Figure 4:**
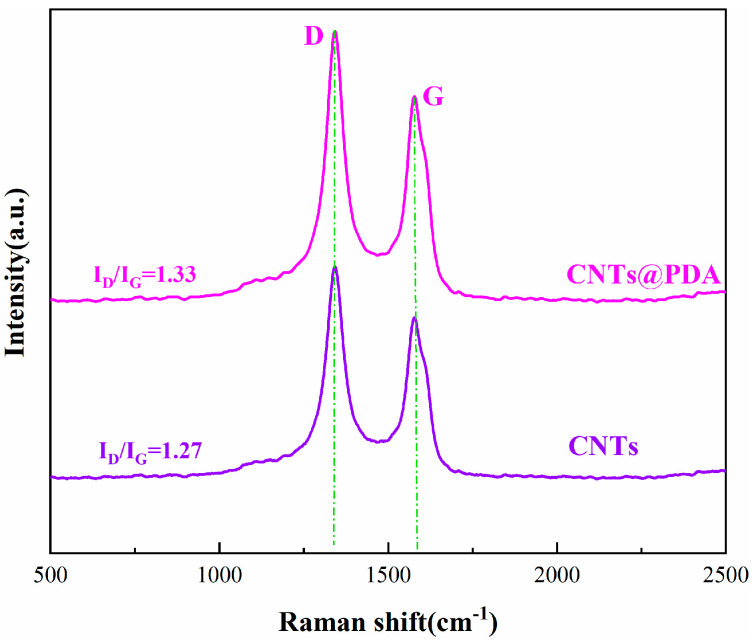
Raman spectra of CNTs and CNTs@PDA.

Raman spectroscopy can distinguish between the hybrid forms of carbon atoms and is also an effective method to examine the structural characteristics of CNTs. The Raman spectra of the CNTs and the CNTs@PDA were both double peaks, as shown in Figure 4. The Raman spectra of CNTs showed a D band (1334 cm^−1^) and a G band (1581 cm^−1^), indicating that the graphitic structure was preserved in CNTs and CNTs@PDA [39]. If R is defined as the intensity ratio of peak D to peak G, the value of R can reflect the functionalization degree and the integrity of the CNTs graphite structure [40]. After calculation, the R values of the CNTs and the CNTs@PDA were 1.27 and 1.33, respectively. In contrast, the R value of the CNTs@PDA was only slightly greater than that of the CNTs, which was due to the introduction of extra heteroatoms (N), resulting in the decomposition of the graphitic structure, producing more defects and disordered areas within CNTs [41].

**Figure 5:**
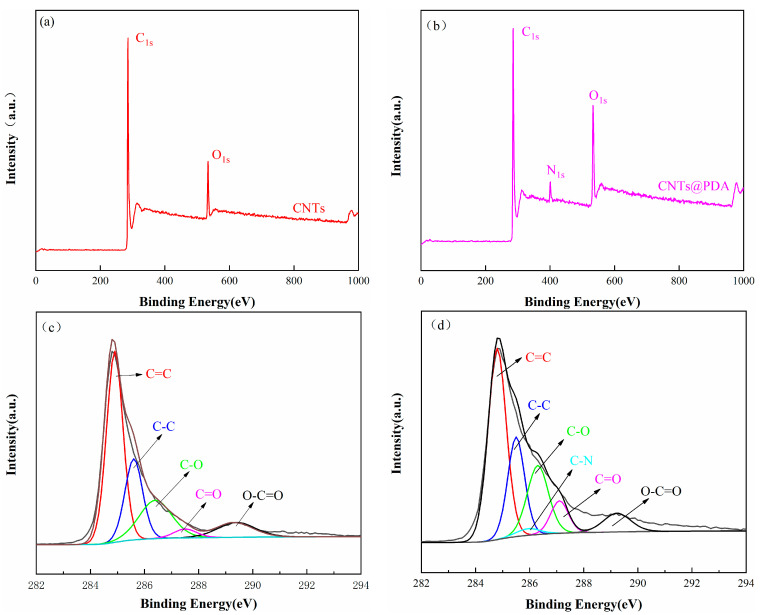
(**a**) XPS survey spectra of CNTs and (**b**) CNTs@PDA; C1s XPS spectra of (**c**) CNTs and (**d**) CNTs@PDA.

XPS is an effective means of detecting the surface composition, chemical state, and chemical bond information of materials. The XPS curves and related data are shown in Figure 5 and Table 1, respectively. Figure 5a,b shows the full XPS spectrum of the CNTs and the CNTs@PDA, in which the CNTs and the CNTs@PDA could be seen in the two groups of main peaks corresponding to the C1s and the O1s near 284 and 533 eV. In addition to the C1s peak of 284 eV and the O1s peak of 533 eV in the full spectrum of the CNTs@PDA, the N1s peak also appeared near 400 eV, which confirmed the existence of the PDA. Five sub-peaks (as shown in Figure 5c) were obtained by fitting the C1s peak of the CNTs to the corresponding C=C (284.8 eV), C-C (285.5 eV), C-O (286.4 eV), C=O (287.5 eV), and O-C=O (289.3 eV). As a result, five sub-peaks (as shown in Figure 5c) were obtained, corresponding to C=C (284.8 eV), C-C (285.5 eV), C-O (286.4 eV), C=O (287.5 eV), and O-C=O (289.3 eV). The C1s peak of the CNTs@PDA was analyzed (as shown in Figure 5d), and six sub-peaks were obtained, which were attributed to C=C (284.8 eV), C-C (285.5 eV), C-N (285.9 eV), C-O (286.5 eV), C=O (287.3 eV), and O-C=O (289.2 eV). The new peak of the modified CNTs near 285.9 eV corresponded to the unique N-element on the PDA, which was consistent with the results of the full spectrum in Figure 5d. All of the above results indicate that the PDA successfully coated the CNTs.

**Figure 6:**
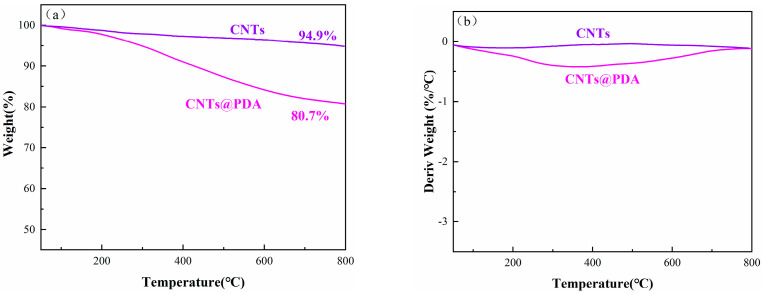
TGA (**a**) and DTG (**b**) curves of CNTs and CNTs@PDA.

The thermal stability of the CNTs@PDA was characterized by thermogravimetric analyzer. Figure 6 shows the thermogravimetric curves of the CNTs and the CNTs@PDA. As shown in the figure, the unmodified CNTs did not show significant weight loss during the whole thermal analysis process, which was in line with the excellent heat resistance characteristics of carbon nanomaterials. While the CNTs@PDA had begun to show weight loss at 250 °C, they had an obvious weight loss between 300 and 600 °C, which was mainly due to the thermal decomposition of the polydopamine. When the temperature increased to 800 °C, the thermal weight loss of the CNTs@PDA accounted for about 19.3% of the total weight, showing that the PDA had been successfully coated on the surface of the CNTs.

### 3.2. Crystallization Properties

**Figure 7:**
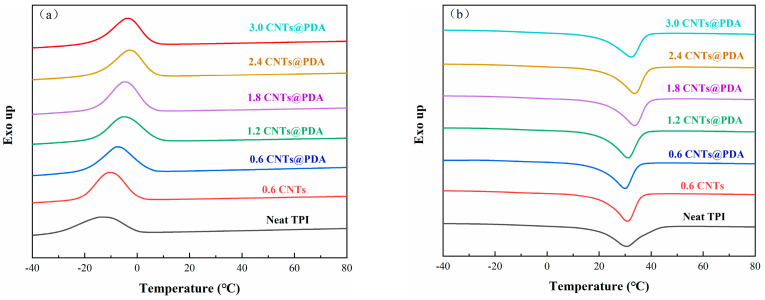
DSC curves of TPI composites: (**a**) cooling curves and (**b**) heating curves.

The crystallization and crosslinking structure of TPI plays an important role in its mechanical and shape-memory properties. We used DSC to characterize the crystallization and melt-transition characteristics of TPI composites. The DSC curves and related data are shown in Figure 7 and Table 2, respectively. The results show that the melting temperature (T_m_), crystallization temperature (T_c_), melting enthalpy (∆H_m_), and crystallinity (X_c_) of the TPI/CNTs@PDA composites were higher than those of neat TPI and TPI/CNTs. The reason was that the agglomeration of neat CNTs in the matrix hindered the crystallization of the TPI molecular chains and led to the decrease in the crystallinity of the materials [42]. The results also indicate that the CNTs@PDA had better dispersion in the TPI composites. It is worth noting that the crystallinity of the composites first increased and then decreased with the additional amount of the CNTs@PDA. Two possibilities could explain this phenomenon: On the one hand, the CNTs@PDA may have acted as a nucleating agent in the composites, which could have promoted the crystallization process of the chain segment. On the other hand, when the total amount of the CNTs@PDA reached a certain temperature, the strong interaction between the CNTs and the polymer greatly reduced the regularity of the TPI molecular chain, which was not conducive to its crystallization, resulting in a decrease in the TPI crystallinity.

**Figure 8:**
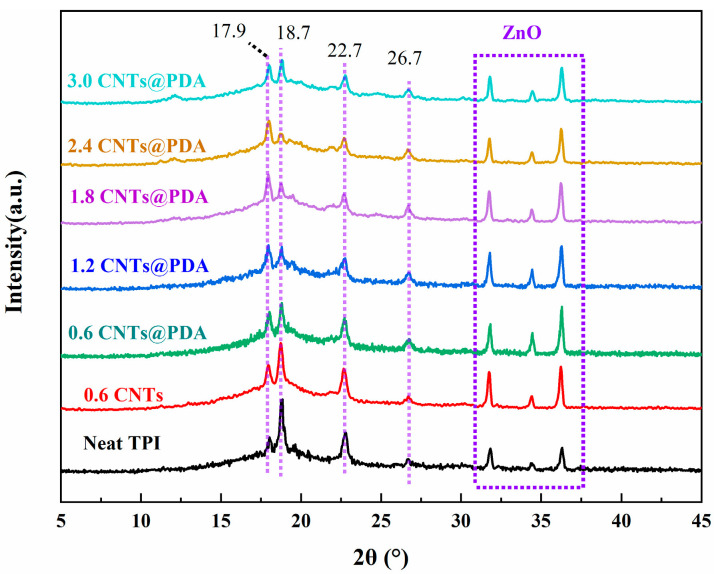
XRD profiles of TPI Composites.

The TPI was a partially crystalline polymer material, which had two crystal forms: *α* and *β*. As shown in the XRD pattern in Figure 8, there were obvious characteristic peaks in the TPI composite material, of which the XRD diffraction angles (2θ) of the *α* and *β* crystal forms were 17.9 (*α*), 26.7 (*α*), 18.7 (*β*), and 22.7 (*β*) [43]. The three peaks between 30° and 40° corresponded to the characteristic peaks of ZnO [44]. In addition, there were no new crystallization peaks in the XRD curves of all the samples, which indicated that the crystal structure of the TPI composites had not been destroyed.

### 3.3. Thermal Properties

**Figure 9:**
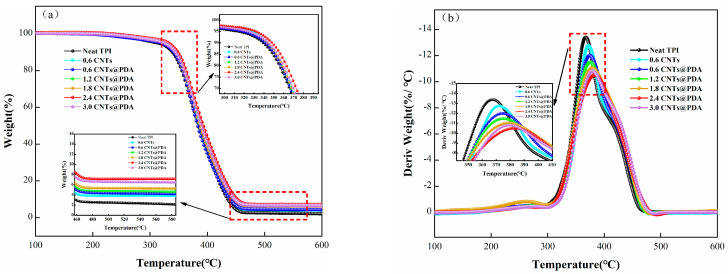
TGA (**a**) and DTG (**b**) analysis of TPI composites.

The TGA and DTG curves of the sample are shown in Figure 9, while the main characteristic parameters, such as the initial degradation temperature (T_5%_), the maximum thermal degradation temperature (T_max_) and carbon residue at 600 °C, are summarized in Table 3. Under a nitrogen atmosphere, the TPI/CNTs@PDA system had a higher T_5%_, T_max_, and carbon residue than the neat TPI and TPI/CNTs systems. With the increase in CNTs@PDA content, T_5%_, T_max_ and carbon residue of TPI composites were increased by varying degrees. This could be attributed to the fact that polydopamine has a large number of benzene rings and hydroxyl structures. A large number of hydroxyl groups means crystalline water is formed more easily, and since the polymer loses water during combustion, it reduces the temperature as well. At the same time, a rich benzene ring structure can form carbon with high thermal stability that can then accumulate on the surface of the TPI matrix and form a dense carbon layer. As a thermal insulation layer, the thicker carbon layer may not only slow down the process of thermal degradation but may also prevent heat transfer to the interior of the remaining composite materials. The complementary interaction of the dense carbon layer structure and thermal insulation barrier yielded excellent thermal stability, and they acted upon the TPI/CNTs@PDA composites.

The DTG curve showed that the CNTs@PDA not only promoted the formation of carbon but also slowed down the mass loss rate of the TPI composites during their degradation at high temperatures. The further decomposition of the matrix was delayed, and the carbon yield was increased, which had a positive effect on the thermal stability of the composites.

### 3.4. Mechanical Properties 

**Figure 10:**
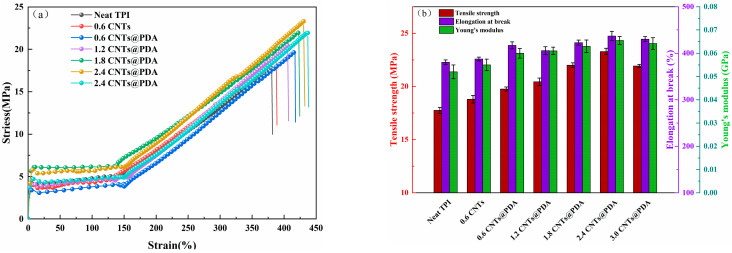
(**a**) Stress–strain curves; (**b**) average stress, elongation-at-break and Young’s modulus of TPI composites with various CNTs@PDA hybrid loadings.

The tensile strength properties of TPI composites at room temperature are shown in Figure 10. The tensile strength, elongation-at-break and Young’s modulus of neat TPI were 17.7 MPa, 380.5% and 0.052 GPa, respectively. The tensile strength, elongation-at-break and Young’s modulus of the composites were increased to 18.8 MPa, 387.6% and 0.055 GPa, respectively, by adding 0.6 phr of the CNTs, which was mainly due to the unique mechanical and physical properties of CNTs, as well as their high specific surface area and aspect ratio [45,46]. When the same amount of CNTs@PDA (0.6 phr) was added, the tensile strength, elongation-at-break and Young’s modulus of the composites were 19.8 MPa, 416.9% and 0.06 GPa, respectively, which showed a better strengthening effect, as compared to the CNTs alone. This was due to the fact that the van der Waals force interaction between the CNTs was relatively strong and was easy to agglomerate, which made the distribution of the CNTs in the TPI matrix uneven, and the agglomerates formed small defects in the matrix, which reduced the tensile properties of the composites. The dispersion of the CNTs@PDA in the TPI was better, which could effectively avoid the agglomeration phenomenon, so the tensile properties of the TPI composites may prove beneficial. In addition, the tensile strength, elongation-at-break and Young’s modulus of the composite reached the highest values when the CNTs@PDA content was 2.4 phr, which were 31.64%, 13.07% and 0.066 GPa higher, respectively, than that of the neat TPI. The tensile properties of the TPI composites improved with the addition of the CNTs@PDA under high load, which was a result of the CNTs@PDA thorough dispersion in the TPI and the strong interfacial bonding between the CNTs and the TPI matrix.

**Figure 11:**
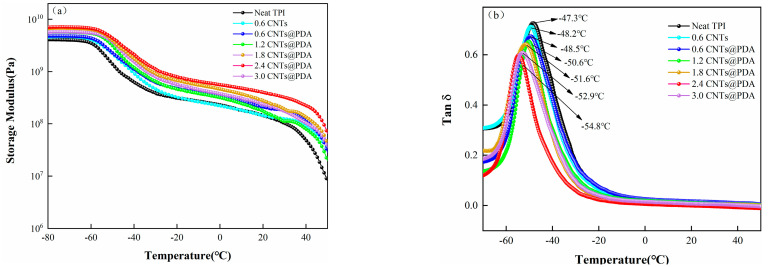
DMA curves of (**a**) storage modulus (E′) and (**b**) loss factor (tan δ) of TPI composites.

The DMA curves of the storage modulus (E′) and the loss factor (tan δ) versus the temperature of the samples are shown in Figure 11. Below the glass transition temperature (T_g_), the addition of CNTs increased the storage modulus (E’) of the TPI composite, as shown in Figure 11a. This result is associated with the hindered movement of the cross-linked molecular chain when CNTs were added into the TPI matrix. In addition, due to the high rigidity of the CNTs, they acted as a skeleton, which led to the improvement of the rigidity of the composites and was reflected by the increased energy storage modulus. As compared to the TPI/CNTs, the E’ of the TPI/CNTs@PDA composites increased when the same content of the CNTs@PDA had been added. This was due to the abundant amounts of amine and catechol groups in the PDA coating layer, which improved the interface interaction between the CNTs@PDA surface and the TPI matrix [47]. However, when the amount of CNTs@PDA exceeded 2.4 phr, the E’ of the composites decreased due to the abundant catechol groups, which not only promoted the dispersions of the CNTs in the TPI matrix but also enabled hydrogen bonding between the PDA coating layers. This resulted in a bridge between the CNTs and, therefore, their agglomeration and resultant defects. These defects formed stress concentration points in the matrix, leading to a decrease in E’. Figure 11b shows that when CNTs@PDA was added to the composites, the T_g_ was −48.5 °C, a reduction of 1.2 °C, as compared to the neat TPI. T_g_ gradually decreased and trended downward when the element content was increased, which in turn increased the distance between the molecular chains and promoted their movement. In addition, with the continuous increase in CNTs@PDA content, the loss factor of the samples tended to decrease, and the loss factor of the samples were lower than that of the neat TPI.

**Figure 12:**
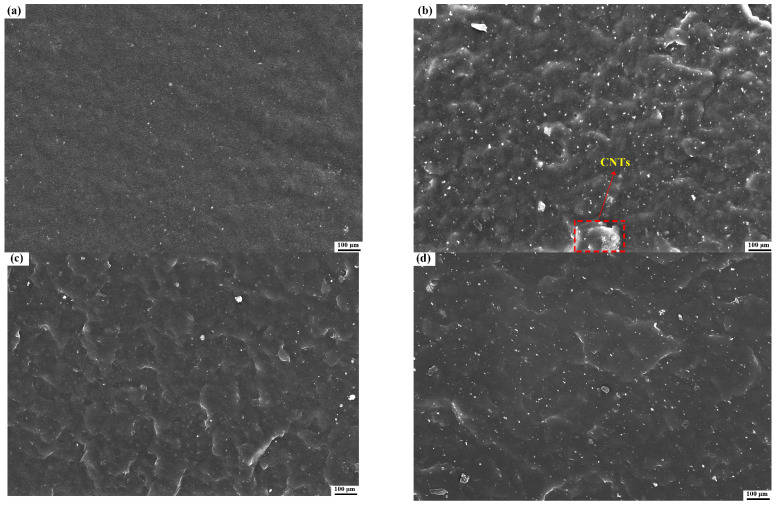

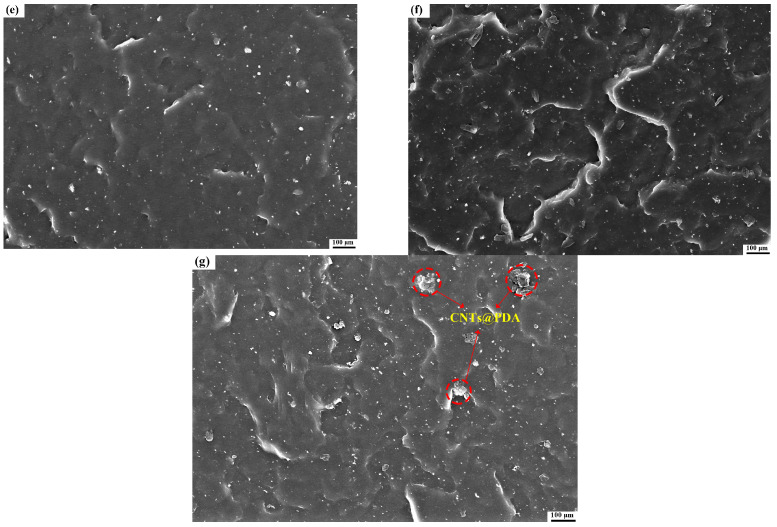
SEM images of fracture sections of TPI composites: (**a**) Neat TPI; (**b**) TPI with 0.6 phr CNTs; (**c**) TPI with 0.6 phr CNTs@PDA; (**d**) TPI with 1.2 phr CNTs@PDA; (**e**) TPI with 1.8 phr CNTs@PDA; (**f**) TPI with 2.4 phr CNTs@PDA; and (**g**) TPI with 3.0 phr CNTs@PDA.

The dispersion state of the nano-fillers in the composites had an impact on their mechanical properties. To further study the dispersion and aggregation of the CNTs@PDA in the TPI, the sectional morphology of the composite was investigated with SEM. The cross-section of the neat TPI was relatively flat and smooth with typical brittle fracture characteristics. Figure 12b shows that the CNTs had a large-scale aggregation, and the cross-section became coarser, as compared to that of the neat TPI. Even though they had the same additional contents, the cross-section of the TPI/CNTs@PDA composites had no aggregation, indicating that the PDA further improved the dispersion of the CNTs in the TPI matrix. In addition, with the gradually increased CNTs@PDA content, a large number of small cracks appeared in the composite, and the density and depth gradually increased. This fracture behavior was due to the strong interfacial interaction between the PDA-modified CNTs and the TPI matrix. More effective stress was transferred from the TPI matrix to the CNTs with high modulus, which inhibited the crack propagation in the matrix on a larger scale, leading to the roughness seen in the cross-section of the composites. The addition of the PDA-coated CNTs improved the fracture toughness of the TPI matrix. However, when the content of CNTs@PDA exceeded 2.4 phr, there was still some small-scale aggregation, and the crack density decreased to a certain extent, as shown in Figure 12g. The suggested cause for this could be that with the increased packing content, the CNTs coated with PDA could still have had a strong association with each other, which could have led to their weak dispersion in the TPI matrix.

### 3.5. Shape-Memory Properties

**Figure 13:**
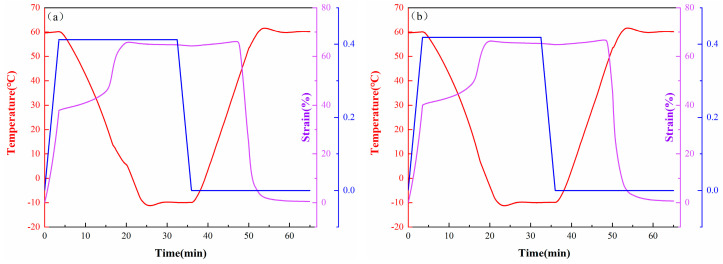

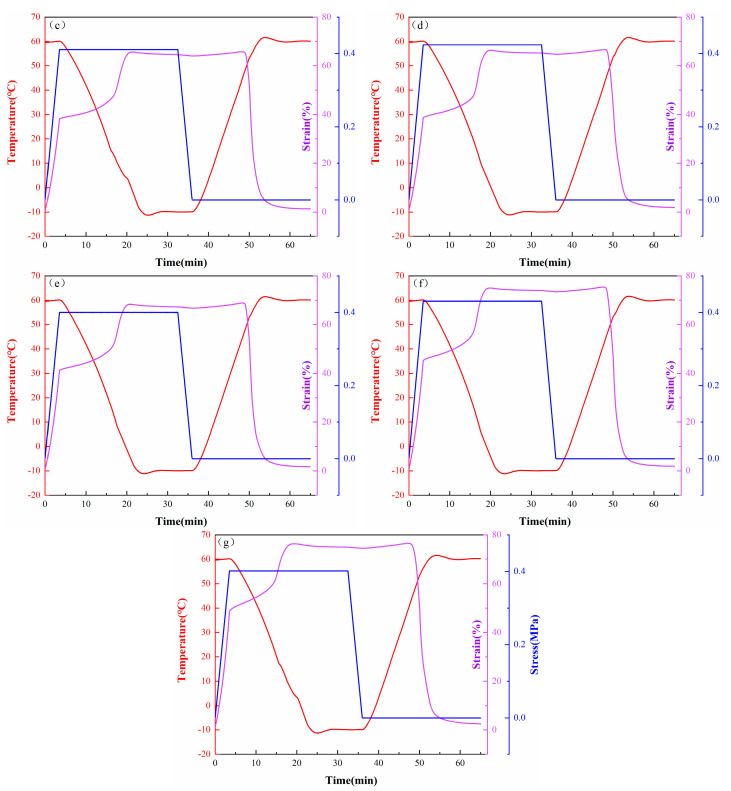
Shape-memory properties of TPI composites: (**a**) Neat TPI; (**b**) TPI with 0.6 phr CNTs; (**c**) TPI with 0.6 phr CNTs@PDA; (**d**) TPI with 1.2 phr CNTs@PDA; (**e**) TPI with 1.8 phr CNTs@PDA; (**f**) TPI with 2.4 phr CNTs@PDA; and (**g**) TPI with 3.0 phr CNTs@PDA.

The shape-memory properties of the samples were measured with DMA, and the results are shown in Figure 13 and Table 4. Because the TPI nanocomposites have excellent shape-memory properties, there was little difference in the *R_f_* values among the samples, and the *R_f_* values of all the samples were maintained at a high level. In addition, the *R_r_* of the neat TPI was 99.8%, and the *R_r_* of the TPI composite with 0.6 phr of the CNTs and the TPI composite with 0.6 phr of the CNTs@PDA decreased to 98.2% and 97.9%, respectively. The reason for this phenomenon was that the CNTs modified with PDA can effectively promote the crystallization of the TPI matrix. This indicated that the elastic recovery of the TPI decreased as the relative content of the TPI cross-linked networks (at the same density) decreased. Nevertheless, the *R_r_* values of all the samples were still greater than 96%, indicating that the TPI composites had excellent shape-recovery properties. These results indicate that the TPI composites had excellent shape-memory performance.

## 4. Conclusions

In this study, we prepared CNTs@PDA nano-fillers via a non-covalent bonding method and then combined it with TPI via a melt-blending method to prepare new shape-memory polymer composites. The effects of different components on the thermal, mechanical, and shape-memory properties of the composites were studied. The SEM results confirm that PDA nanoparticles uniformly coated the surfaces of the CNTs. FTIR, Raman, and XPS spectra were used to determine the chemical structure of the CNTs@PDA. In addition, according to the DSC, XRD, and TGA test results, the crystallization properties and the thermal stability of the samples containing the CNTs@PDA were improved, as compared to those containing only CNTs. Furthermore, the surface and fracture morphologies of the samples were evaluated by SEM, and the results show that PDA could significantly improve the interfacial compatibility of CNTs and TPI, which also improved the mechanical properties of the composites. At the same time, the CNTs@PDA (2.4 phr) composite had the best mechanical properties and shape-memory performance.

## Figures and Tables

**Table 1:** Element content of CNTs and CNTs@PDA.

	O 1s (at.%)	N 1s (at.%)	C 1s (at.%)
CNTs	10.92	/	89.08
CNTs@PDA	16.38	4.24	79.38

**Table 2:** DSC crystallization parameters of TPI composites.

Sample	X_c_ (%)	T_c_ (°C)	T_m_ (°C)	∆H_m_ (J/g)
Neat TPI	21.47	−13.22	30.68	40.11
TPI/0.6 CNTs	21.28	−10.68	29.97	39.76
TPI/0.6 CNTs@PDA	22.39	−7.69	30.92	41.83
TPI/1.2 CNTs@PDA	22.66	−5.55	31.12	42.32
TPI/1.8 CNTs@PDA	25.28	−4.96	33.69	47.23
TPI/2.4 CNTs@PDA	25.62	−3.28	34.12	47.86
TPI/3.0 CNTs@PDA	21.69	−3.88	32.43	42.53

**Table 3:** TGA parameters of TPI composites.

Sample	T_5%_ (℃)	T_max_ (°C)	Weight 600 °C (wt%)
Neat TPI	318.7	380.2	2.5
TPI/0.6CNT	322.1	381.8	4.1
TPI/0.6CNTs@PDA	324.3	382.5	4.2
TPI/1.2CNTs@PDA	326.5	387.6	4.4
TPI/1.8CNTs@PDA	329.5	389.6	4.9
TPI/2.4CNTs@PDA	331.1	392.5	6.8
TPI/3.0CNTs@PDA	326.6	384.8	6.5

**Table 4:** Shape-memory performance parameters for TPI composites.

Samples	*R_f_* (%)	*R_r_* (%)
Neat TPI	97.6	99.8
TPI/0.6CNTs	97.3	98.2
TPI/0.6CNTs@PDA	97.5	97.9
TPI/1.2CNTs@PDA	97.5	97.7
TPI/1.8CNTs@PDA	97.7	97.5
TPI/2.4CNTs@PDA	97. 3	97.4
TPI/3.0CNTs@PDA	97.6	96.7

## Data Availability

Data is contained within the article.

## References

[1] Zhang F., Xia Y., Liu Y., Leng J. (2020). Nano/microstructures of shape memory polymers: From materials to applications. Nanoscale Horiz..

[2] Yu X., Wen C., Zeng W., Zhao S., Wang L., Wan G., Huang S., Grover H., Chen Z. (2018). Mechanical behaviors and biomedical applications of shape memory materials: A review. AIMS Mater. Sci..

[3] Xia Y., He Y., Zhang F., Liu Y., Leng J. (2021). A review of shape memory polymers and composites: Mechanisms, materials, and applications. Adv. Mater..

[4] Peng K., Zhao Y., Shahab S., Mirzaeifar R. (2020). Ductile shape-memory polymer composite with enhanced shape recovery ability. ACS Appl. Mater. Interfaces.

[5] Lendlein A., Gould O.E.C. (2019). Reprogrammable recovery and actuation behaviour of shape-memory polymers. Nat. Rev. Mater..

[6] D’Elia E., Ahmed H.S., Feilden E., Saiz E. (2019). Electrically-responsive graphene-based shape-memory composites. Appl. Mater. Today.

[7] Maiti B., Abramov A., Franco L., Puiggalí J., Enshaei H., Alemán C., Díaz D.D. (2020). Thermoresponsive shape-memory hydrogel actuators made by phototriggered click chemistry. Adv. Funct. Mater..

[8] Chen H.-M., Wang L., Zhou S.-B. (2018). Recent progress in shape memory polymers for biomedical applications. Chin. J. Polym. Sci..

[9] Diaz Lantada A. (2017). Systematic development strategy for smart devices based on shape-memory polymers. Polymers.

[10] Xian J., Geng J., Wang Y., Xia L. (2018). Quadruple-shape-memory effect of TPI/LDPE/HDPE composites. Polym. Adv. Technol..

[11] Yu Z., Wang Z., Li H., Teng J., Xu L. (2019). Shape memory epoxy polymer (SMEP) composite mechanical properties enhanced by introducing graphene oxide (GO) into the matrix. Materials.

[12] Miaudet P., Derre A., Maugey M., Zakri C., Piccione P.M., Inoubli R., Poulin P. (2007). Shape and temperature memory of nanocomposites with broadened glass transition. Science.

[13] Meng Q., Hu J. (2009). A review of shape memory polymer composites and blends. Compos. Part A Appl. Sci. Manuf..

[14] Pras M., Gerard J.F., Golanski L., Quintard G., Duchet-Rumeau J. (2020). Key role of the dispersion of carbon nanotubes (CNTs) within epoxy networks on their ability to release. Polymers.

[15] Zare Y., Rhee K.Y. (2020). Analysis of the connecting effectiveness of the interphase zone on the tensile properties of carbon nanotubes (CNT) reinforced nanocomposite. Polymers.

[16] Sakharova N.A., Pereira A.F.G., Antunes J.M., Fernandes J.V. (2020). Mechanical characterization of multiwalled carbon nanotubes: Numerical simulation study. Materials.

[17] Kuroda C., Haniu H., Ajima K., Tanaka M., Sobajima A., Ishida H., Tsukahara T., Matsuda Y., Aoki K., Kato H. (2016). The dispersion state of tangled multi-walled carbon nanotubes affects their cytotoxicity. Nanomaterials.

[18] Gravely A.A., Cutting A., Nugent S., Grill J., Carlson K., Spoont M. (2011). Validity of PTSD diagnoses in VA administrative data: Comparison of VA administrative PTSD diagnoses to self-reported PTSD Checklist scores. J. Rehabil. Res. Dev..

[19] El-Mageed A.I.A.A., Ogawa T. (2018). Metal ion effect on the supramolecular structures of metalloporphyrins on single-walled carbon nanotube surface. Appl. Surf. Sci..

[20] Hirsch A. (2002). Functionalization of single-walled carbon nanotubes. Angew. Chem. Int. Ed..

[21] Abd El-Mageed A.I.A., Ogawa T. (2019). Supramolecular structures of terbium(iii) porphyrin double-decker complexes on a single-walled carbon nanotube surface. RSC Adv..

[22] Abd El-Mageed A.I.A., Handayani M., Chen Z., Inose T., Ogawa T. (2019). Assignment of the absolute-handedness chirality of single-walled carbon nanotubes using organic molecule supramolecular structures. Chemistry.

[23] Kawamoto M., He P., Ito Y. (2017). Green processing of carbon nanomaterials. Adv. Mater..

[24] Song X., Zhang Y., Wang Y., Huang M., Gul S., Jiang H. (2020). Nanocomposite membranes embedded with dopamine-melanin nanospheres for enhanced interfacial compatibility and nanofiltration performance. Sep. Purif. Technol..

[25] Liu L. (2002). Organic modification of carbon nanotubes. Chin. Sci. Bull..

[26] Nejatian T., Nathwani N., Taylor L., Sefat F. (2020). Denture base composites: Effect of surface modified nano- and micro-particulates on mechanical properties of polymethyl methacrylate. Materials.

[27] Yao D., Yin G., Bi Q., Yin X., Wang N., Wang D.Y. (2020). Basalt fiber modified ethylene vinyl acetate/magnesium hydroxide composites with balanced flame retardancy and improved mechanical properties. Polymers.

[28] Lin Q., Gourdon D., Sun C., Holten-Andersen N., Anderson T.H., Waite J.H., Israelachvili J.N. (2007). Adhesion mechanisms of the mussel foot proteins mfp-1 and mfp-3. Proc. Natl. Acad. Sci. USA.

[29] Nguyen D.N., Sim U., Kim J.K. (2020). Biopolymer-inspired n-doped nanocarbon using carbonized polydopamine: A high-performance electrocatalyst for hydrogen-evolution reaction. Polymers.

[30] Lee H., Dellatore S.M., Miller W.M., Messersmith P.B. (2007). Mussel-inspired surface chemistry for multifunctional coatings. Science.

[31] Lee B.P., Messersmith P.B., Israelachvili J.N., Waite J.H. (2011). Mussel-inspired adhesives and coatings. Annu. Rev. Mater. Res..

[32] Yan B., Zhou Q., Xing T., Chen G. (2018). Dopamine-dyed and functionally finished silk with rapid oxidation polymerization. Polymers.

[33] Bi Q., Yao D., Yin G.-Z., You J., Liu X.-Q., Wang N., Wang D.-Y. (2020). Surface engineering of magnesium hydroxide via bioinspired iron-loaded polydopamine as green and efficient strategy to epoxy composites with improved flame retardancy and reduced smoke release. React. Funct. Polym..

[34] Koh K.L., Ji X., Dasari A., Lu X., Lau S.K., Chen Z. (2017). Fracture toughness and elastic modulus of epoxy-based nanocomposites with dopamine-modified nano-fillers. Materials.

[35] Lin C., Gong F., Yang Z., Zhao X., Li Y., Zeng C., Li J., Guo S. (2019). Core-shell structured hmx@polydopamine energetic microspheres: Synergistically enhanced mechanical, thermal, and safety performances. Polymers.

[36] Song H., Wang Z., Yang J., Jia X., Zhang Z. (2017). Facile synthesis of copper/polydopamine functionalized graphene oxide nanocomposites with enhanced tribological performance. Chem. Eng. J..

[37] Huan X., Shi K., Yan J., Lin S., Li Y., Jia X., Yang X. (2020). High performance epoxy composites prepared using recycled short carbon fiber with enhanced dispersibility and interfacial bonding through polydopamine surface-modification. Compos. Part B Eng..

[38] Xia L., Gao H., Geng J.T. (2019). Facile fabrication of foamed natural Eucommia ulmoides gum composites with heat-triggered shape memory behavior. Polym. Compos..

[39] Pimenta M.A., Dresselhaus G., Dresselhaus M.S., Cancado L.G., Jorio A., Saito R. (2007). Studying disorder in graphite-based systems by Raman spectroscopy. Phys. Chem. Chem. Phys..

[40] Liu J., Chen C., Feng Y., Liao Y., Ye Y., Xie X., Mai Y.W. (2018). Ultralow-carbon nanotube-toughened epoxy: The critical role of a double-layer interface. ACS Appl. Mater. Interfaces.

[41] Liu S., Li G., Gao Y., Xiao Z., Zhang J., Wang Q., Zhang X., Wang L. (2017). Doping carbon nanotubes with N, S, and B for electrocatalytic oxygen reduction: A systematic investigation on single, double, and triple doped modes. Catal. Sci. Technol..

[42] Xia L., Wu H., Qiu G. (2019). Shape memory behavior of carbon nanotube-reinforced trans-1,4-polyisoprene and low-density polyethylene composites. Polym. Adv. Technol..

[43] Niu Q., Jiang X., He A. (2014). Synthesis of spherical trans-1,4-polyisoprene/trans-1,4-poly(butadiene-co-isoprene) rubber alloys within reactor. Polymer.

[44] Li Q., Kumar V., Li Y., Zhang H., Marks T.J., Chang R.P.H. (2005). Fabrication of ZnO nanorods and nanotubes in aqueous solutions. Chem. Mater..

[45] Zanjanijam A.R., Bahrami M., Hajian M. (2016). Poly(vinyl chloride)/single wall carbon nanotubes composites: Investigation of mechanical and thermal characteristics. J. Vinyl. Addit. Technol..

[46] Zanjanijam A.R., Hajian M., Koohmareh G.A. (2014). Improving the thermal and mechanical properties of poly(vinyl butyral) through the incorporation of acid-treated single-walled carbon nanotubes. J. Appl. Polym. Sci..

[47] Ling Y., Li W., Wang B., Gan W., Zhu C., Brady M.A., Wang C. (2016). Epoxy resin reinforced with nanothin polydopamine-coated carbon nanotubes: A study of the interfacial polymer layer thickness. RSC Adv..

